# Perspective: on the future of fecal microbiota transplantation

**DOI:** 10.3389/fmicb.2024.1449133

**Published:** 2024-09-09

**Authors:** Olaf F. A. Larsen, Robert J. M. Brummer

**Affiliations:** ^1^Athena Institute, Vrije Universiteit Amsterdam, Amsterdam, Netherlands; ^2^Faculty of Medicine and Health, School of Medical Sciences, Nutrition-Gut-Brain Interactions Research Centre, Örebro University, Örebro, Sweden

**Keywords:** fecal microbiota transplantation, gut microbiota, probiotics, personalized, analogs, One Health

## Abstract

Fecal Microbiota Transplantation (FMT) has shown to possess impressive potential benefit for a wide range of clinical indications. Due to its inherent safety issues and efficacy constraints, the use of personalized FMT analogs could be a promising avenue. The development of such analogs will require a detailed understanding of their functionality, encompassing not only microbe-host interactions of the microbial taxa that are involved, but also of the ecological dimensions of the analogs and an overview of the gastrointestinal sites where these relevant microbial interactions take place. Moreover, characterization of taxa that have been lost due to diminished exposure to beneficial microbes, as a consequence of Western lifestyle, may lead to creation of future FMT analogs with the capacity to restore functionalities that we have lost.

## 1 Introduction

Fecal microbiota transplantation (FMT) encompasses the transfer of fecal matter from a healthy donor to a recipient in order to restore its gut microbial ecosystem with the aim to prevent, treat or alleviate a clinical disorder. The technique has already been described in the fourth century in China, in which a human fecal suspension was orally administered to patients suffering from food poisoning or severe diarrhea (Zhang et al., 2012). In modern times, the first published FMT treatment originated from 1958, when it was successfully applied to four patients suffering from pseudomembranous colitis (Halkjær et al., 2023). It is known that pseudomembranous colitis is caused by infection with *Clostridioides difficile* (*C. diff*.) bacteria. After the landmark paper by Van Nood et al. (2013), in which FMT was shown to be highly efficacious for the treatment of recurrent *C. diff*. infections, the technique has drawn huge attention. In general, heterologous transplantation is commonly utilized, however autologous transplantations have also been reported, for example to boost microbial diversity after antibiotic treatment (Taur et al., 2018). A plethora of studies investigating the potential of FMT for various disorders have been published, ranging from Irritable Bowel Syndrome (IBS) (Wang et al., 2023), to Inflammatory Bowel Disease (Imdad et al., 2023) and autism spectrum disorder (Zhang et al., 2023). Currently, the potential of FTM to improve tumor immunotherapy by remodeling the gut microbiota toward a composition resulting in a more favorable efficacy of immune checkpoint inhibitors, has been recognized as well (Yang et al., 2024). Despite its promising potential, FMT suffers from an inherent safety risk, as transfer of non-pathogenic microorganisms only cannot be warranted. Furthermore, FMT is not as a standardized treatment compared to conventional pharmaceutical therapy, as the composition of the stool is inherently dependent on the donor. This has initiated an intense debate whether FMT should be regarded as tissue transplantation or as pharmacological treatment which has substantial consequences for its research. Classifying conventional FMT as a pharmacologic treatment would infer severe limitations to its research. Consequently, the development of defined consortia is an area of intense research, with recently two products being approved by the FDA for the eradication of *C. diff*. (FDA website). However, treatment of other disorders than specifically related to *C. diff*. Probably need intervention with more complex microbiological ecosystems to be successful. As the gut microbiota is known to be unique for every individual, it is to be expected that the number needed to treat for future successful FTM applications will be (much) higher than that for the eradication of a single pathogen. A study by Kootte et al. (2017), in which FMT was being investigated to improve insulin sensitivity among patients suffering from metabolic syndrome, hinted already in this direction (Kootte et al., 2017). In this study, it was shown that efficacy was correlated to the baseline fecal microbiota composition of the patient. A recent study also reports on the drivers determining the efficacy of FMT colonization (Schmidt et al., 2022). Hence, to improve efficacy of future FMT products, a more personalized approach seems to be necessary. In this perspective, we will touch upon factors that should be taken into account for the development of such future rationalized products.

## 2 On the ecological dimensions of future FMT analogs

FMT represents a “bulk approach,” in which the total gastrointestinal microbiota of a donor is being transferred to a patient. As the aim of such a transfer is the clearance or alleviation of a clinical disorder, it seems plausible, instead, only to transfer the microbes needed to treat that specific pathologic condition. As such, one would like to know which specific functionality (or functionalities) is (are) lost or malfunctioning. This can be due to a lack or low abundance of keystone taxa. Keystone taxa act as “interactional hubs” within a microbial network, and are known to be crucial for the structure of such networks (Banerjee et al., 2018). Absence of keystone taxa will result in a structural collapse of the microbial network it makes part of and consequently its associated functionality. Alternatively, a lack or low abundance of microbial guilds can be the causing of the pathophysiologic condition. A microbial guild is a small ecosystem, comprising a limited number of taxa that work together, and dedicated to a single specific functionality (Wu et al., 2021). It has been shown that such microbial guilds indeed exists in the human gastrointestinal tract and are, among others, involved in the etiology of type 2 diabetes mellitus (Zhang et al., 2015; Zhao et al., 2018). Hence, a future FTM analog may comprise such keystone taxa and/or microbial guilds. Earlier mathematical modeling on such small ecosystems demonstrated that a higher richness of taxa will contribute to both stability and efficiency of the process the guild is dedicated to Larsen and Claassen (2018). In order to produce a stable FTM analog, such preparations should consist of networks comprising at least five to six different taxa (Larsen et al., 2019).

## 3 On the microbial composition of future FMT analogs

Currently, most research on the composition of the gut microbiota is still focusing on the bacterial composition, the so-called bacteriome. This is not surprising, knowing that the number of gut-bacteria (≈ 4 × 10^13^) roughly equals the total number of somatic cells (Sender et al., 2016). However, other domains present in the intraluminal intestinal ecosystem also play an essential role in maintaining homeostasis or causing pathophysiological aberrations in the body. For example, the archea (collectively designated as the archeome) have been mapped (Chibani et al., 2022) and recent evidence suggests a synergistic metabolic relationship between the bacterium *Bacteroides thetaiotaomicron* and the archeon *Methanobrevibacter smithii* (Catlett et al., 2022). Both microorganisms are known to play important roles in health, suggesting that the crosstalk between different domains has to be understood to adequately develop FTM analogs. *Methanobrevibacter smithii* levels are shown to be increased in constipation-predominant IBS (Chong et al., 2019). Hence, one needs knowledge on these types of interdomain crosstalk to effectively treat IBS using FMT analogs, instead of solely focusing on the bacteriome. Similar arguments can be made for the phageome, which can be considered as a subset of the virome, of which its interactions with the bacteriome alter the functional activities of the bacteria (Cao et al., 2022; Kirk et al., 2024). On top of this, the collection of parasites, the parasitome (Ianiro et al., 2022), will undoubtedly also play an important role, as it is known to influence the immune system (Ianiro et al., 2022). In addition to these different microbial domains, microbial components and the produced microbial metabolites may also play a role in the overall efficacy of FMT, which was suggested by a study in which a sterile FMT showed to be sufficient to effectively treat patients suffering from *C. diff*. Infection (Ott et al., 2017). A study by Paramsothy et al. (2019) indeed showed that short-chain fatty acids and secondary bile acids were instrumental for the efficacy of FMT for the treatment of ulcerative colitis (Paramsothy et al., 2019). Hence, future FMT analogs will most likely not only consist of bacteria, but of taxa from different domains, possibly enriched with postbiotics.

## 4 On the mode of action of future FTM analogs

The intraluminal ecosystem is generally used as a proxy for the microbial composition and associated diversity (Tang et al., 2020). As such, fecal samples can be used. However, it is well known that human colonic microbiota interact with the intestinal mucosa both in a healthy as well as in pathological conditions (ulcerative colitis, post infectious IBS, rheumatoid arthritis, among others) and triggers immune-responses (Bachmann et al., 2022; Holster et al., 2020; Holster et al., 2019). As it is known that the microbial composition of the mucosa-associated microbiota significantly differs from its luminal counterpart (Holster et al., 2019; Rangel et al., 2015), it is of importance that the composition of a possible FMT analog also adequately matches with this mucosa-associated ecosystem and the intestinal immune system in a functional perspective. In case of an inadequately overstimulated mucosal immune response, such as could be hypothesized in post-infectious IBS, the aim of FMT would be normalizing this immune response rather than changing microbe-microbe interactions or metabolic effects. In ulcerative colitis or microscopic colitis, however, increased production and mucosal utilization of the microbial metabolite butyrate could also be the therapeutic target. The route of administration of FMT analogs should also be considered in relation to its composition and potential mode-of-action. Proximal small bowel administration will trigger a substantially stronger and qualitative different immune response compared to applying the traditional colonic administration. Moreover, the gut microbial composition gradually changes (among others, from a more aerobic to a more anaerobic nature) along the gastrointestinal tract (Hillman et al., 2017). Hence, there are various reasons why an FMT analog should be tailored to the specific location its anticipated action should take place.

## 5 Discussion

In general, the development of future FTM analogs requires a highly detailed functional understanding of how homeostasis and dysbiosis maintain health or cause disease. Depending on the complexity of these (patho)physiological processes one aims to intervene, this understanding may be on the level of generic features at a population level for relatively “simple” disorders, like recurrent *C. diff*. Infections. Alternatively, this knowledge should be at a personalized level like for neurological indications. As such, the comparison can be made with probiotics, for which its therapeutic target ranges from the prevention of antibiotic-associated diarrhea, which is applicable to many probiotics, up to very specific effects that are strain specific (Hill et al., 2014). This detailed understanding will not only encompass all microbial domains involved, but also all sites where possible microbe-microbe and microbe-host interactions may take place, which is clearly not limited to the lumen only. Furthermore, we argue that future FTM analogs ultimately require a One Health approach. Our Western lifestyle with, among others, high antibiotics usage, less direct contact to nature and a low fiber diet, has brough us close to the point of a “catastrophic collapse” in which we are losing functionalities due to a strongly diminished microbial repertoire (Larsen and van de Burgwal, 2021). A detailed understanding of the interactions between the human species and its adjacent ecosystems like soil, plants, and animals, each harboring its own unique microbiota, may lead to the identification of key taxa and/or guilds that may be used to restore functionalities in our body that were lost already, or for reinforcement of such functionalities. To conclude, future FTM analogs may not only be used to alleviate or even cure indications on a personalized level, but also could be applied to (partly) restore the taxa and associated functionalities we have lost as a consequence of our Westernized lifestyle.

## Data Availability

The original contributions presented in the study are included in the article/supplementary material, further inquiries can be directed to the corresponding author.
